# Levels of Protein C and Soluble Thrombomodulin in Critically Ill Patients with Acute Kidney Injury: A Multicenter Prospective Observational Study

**DOI:** 10.1371/journal.pone.0120770

**Published:** 2015-03-19

**Authors:** Josée Bouchard, Rakesh Malhotra, Shamik Shah, Yu-Ting Kao, Florin Vaida, Akanksha Gupta, David T. Berg, Brian W. Grinnell, Brenda Stofan, Ashita J. Tolwani, Ravindra L. Mehta

**Affiliations:** 1 Division of Nephrology and Hypertension, Department of Medicine, University of California San Diego, San Diego, California, United States of America; 2 Division of Nephrology, Department of Medicine, Université de Montréal, Montréal, Canada; 3 Division of Biostatistics and Bioinformatics, University of California San Diego, San Diego, California, United States of America; 4 Biotechnology Discovery Research, Lilly Research Laboratories, Lilly Corporate Center, Indianapolis, United States of America; 5 Division of Nephrology, University of Alabama, Birmingham, Alabama, United States of America; University of Louisville, UNITED STATES

## Abstract

Endothelial dysfunction contributes to the development of acute kidney injury (AKI) in animal models of ischemia reperfusion injury and sepsis. There are limited data on markers of endothelial dysfunction in human AKI. We hypothesized that Protein C (PC) and soluble thrombomodulin (sTM) levels could predict AKI. We conducted a multicenter prospective study in 80 patients to assess the relationship of PC and sTM levels to AKI, defined by the AKIN creatinine (AKI Scr) and urine output criteria (AKI UO). We measured marker levels for up to 10 days from intensive care unit admission. We used area under the curve (AUC) and time-dependent multivariable Cox proportional hazard model to predict AKI and logistic regression to predict mortality/non-renal recovery. Protein C and sTM were not different in patients with AKI UO only versus no AKI. On intensive care unit admission, as PC levels are usually lower with AKI Scr, the AUC to predict the absence of AKI was 0.63 (95%CI 0.44-0.78). The AUC using log10 sTM levels to predict AKI was 0.77 (95%CI 0.62-0.89), which predicted AKI Scr better than serum and urine neutrophil gelatinase-associated lipocalin (NGAL) and cystatin C, urine kidney injury molecule-1 and liver-fatty acid-binding protein. In multivariable models, PC and urine NGAL levels independently predicted AKI (p=0.04 and 0.02) and PC levels independently predicted mortality/non-renal recovery (p=0.04). In our study, PC and sTM levels can predict AKI Scr but are not modified during AKI UO alone. PC levels could independently predict mortality/non-renal recovery. Additional larger studies are needed to define the relationship between markers of endothelial dysfunction and AKI.

## Introduction

In critically ill patients, acute kidney injury (AKI) portends a high mortality rate [1] and is usually detected based on serum creatinine (Scr) levels several hours after injury has occurred. More recently changes in urine output (UO) have been recognized as early markers of renal functional changes [2]. However, both Scr and UO are not specific with respect to the etiology and underlying pathophysiology of AKI. Several biomarkers are now being studied to facilitate an early recognition of AKI and to better define its course [3–8]. Most observational studies have focused on well-established markers of AKI however few have evaluated the contribution of more systemic markers of organ dysfunction [9,10]. Endothelial dysfunction is an early event which has been implicated in the pathophysiology of several conditions including sepsis, ischemia-reperfusion injury and acute lung injury [11]. Markers of endothelial dysfunction and inflammation such as Protein C (PC), soluble thrombomodulin (sTM) and interleukin-6 can be altered in these conditions prior to clinical organ dysfunction [12,13] and may add prognostic value. Experimental studies have shown that AKI represents a systemic disorder where endothelial dysfunction is an early event [14].

There are however very limited data on the role of endothelial changes in clinical AKI. In a single-center study of surgical critically ill patients, PC levels were inversely correlated with organ failure [9]. Another study has shown that elevated sTM levels at hospital admission in trauma patients increase the risk of AKI [10]. No clinical data are available on sTM levels in AKI. The aim of our study was to investigate the evolution of PC and sTM concentrations and their relationship to AKI in a cohort of critically ill patients. We hypothesized that there are derangements in sTM and Protein C levels in AKI and that these alterations can help in the prediction and differential diagnosis of AKI. We also compared the performance of these biomarkers to more commonly used biomarkers to predict AKI, and assessed their performance to predict mortality and non-renal recovery.

## Material and Methods

### Study setting and study participants

We conducted a multicenter prospective observational study on PC and sTM levels in critically ill patients at risk for AKI. Patients were screened at intensive care unit (ICU) admission at three academic medical centers (University of California San Diego (UCSD), University of Alabama and Université de Montréal). Patients were eligible if they were aged 18 or older and had a life expectancy of at least 48 hours. Patients were excluded if they had AKI based on AKIN Scr criterion [15], were admitted to the ICU >48 hours prior to screening, transferred from another ICU, had a Scr >177 μmol/l within three days before ICU admission, were prisoners, received dialysis within 12 months, had a kidney transplant, were on anticoagulants (intravenous unfractionated heparin, low-molecular weight heparin, or warfarin) within the last 7 days, suffered from decompensated cirrhosis, had chronic kidney disease (CKD) stage 5, were anemic (hemoglobin <90 g/l or hematocrit <27%) or enrolled in another research project. The Institutional Review Boards of UCSD, University of Alabama and Université de Montréal approved the study and written informed consent was obtained from all participants or their surrogates prior to their inclusion in the study. Patients with surrogate consents were reconsented if they regained decision-making capacity. The study protocol was in adherence with the Declaration of Helsinki.

### Data Collection

Data on past medical history were collected once, and clinical, laboratory and process-of-care elements were collected daily. AKI was defined as an increase in Scr level of more than 27 μmol/l or 50% from a reference creatinine within 48 hours or UO less than 0.5 ml/kg/hour for at least 6 hours (AKIN criteria) [15]. CKD status was determined from history and chart review. We computed estimated glomerular filtration rate with the Modification of Diet in Renal Disease (MDRD) formula [16]. Acute Physiology and Chronic Health Evaluation (APACHE) III [17] and Sequential Organ Failure Assessment (SOFA) scores [18] were computed daily. Sepsis was assessed daily according to the American College of Chest Physicians/Society of Critical Care Medicine criteria [19]. Outcome measures included mortality, requirement for dialysis and renal function at hospital discharge, and lengths of stay.

### Sample Collection and Processing

Blood samples were collected at study enrollment and every 12 hours for four days. Patients without AKI Scr within the first four days then had blood samples once daily for three days. Patients who developed AKI Scr had blood samples every 12 hours for four days and then daily for another four days, for a maximum of 10 days. Blood was centrifuged at 3500g for 15 minutes, and the supernatant was aliquoted into 0.5 ml fractions and frozen at -70°C.

PC levels were measured within three months at UCSD in EDTA-containing tubes using the protein C chromogenic assay on ACL TOP (Instrumentation Laboratory Company, Lexington, MA). STM levels were measured blinded at Eli Lily and Company within three months in EDTA-containing tubes using the human thrombomodulin ELISA assays and according to the manufacturer’s recommendations using a 1:2 dilution of the plasmas in the kit dilution buffer (Cell Sciences, Canton, MA). All samples were de-identified with respect to patient identifiers and AKI status. PC levels were expressed as a percentage of clotting time with a normal range of 70 to 160%. The within run %CV was 2.6% and the inter-day precision was 3.3%. STM levels were expressed as ng/ml with a normal range of 2.39 to 7.9 ng/ml. The within run %CV was 2.4% and the inter-day precision was 5.5. Neutrophil gelatinase-associated lipocalin (NGAL), cystatin C and kidney injury molecule 1 (KIM-1) were measured using the Meso Scale Discovery (Rockville, Maryland) multiarray platform. Urine liver-fatty acid-binding protein (L-FABP) was measured using an ELISA kit from R&D systems (Minneapolis, Minnesota).

### Statistical analysis

Continuous variables were expressed as mean ± standard deviation or median and interquartile range, where appropriate. Comparison between 2 groups were determined by using *t* test or Mann-Whitney U test, and between more than 2 groups by the one-way ANOVA or Kruskal-Wallis, where appropriate. Categorical variables were expressed as proportions and compared with the Pearson’s *x* ^2^ or Fischer’s exact test, where appropriate. We performed univariable and multivariable time-dependent Cox proportional hazard model to predict AKI, considering these variables: PC, sTM, their interaction, serum and urine NGAL and cystatin C, urine KIM-1 and L-FABP, APACHE III score and sepsis status. To adjust for the biomarkers of interest, the multivariable Cox proportional hazard model was used predicting time to AKI Scr. The model was started with all the biomarkers listing above as predictors, except for serum NGAL due to its correlation with urine NGAL, and the backward selection with 0.20 level p-value was applied to derive the final model. PC and sTM were forced in the model if their interaction term was in the model. In addition, the receiver operating characteristics (ROC) curve analysis was done evaluating the discrimination potential of the biomarkers at ICU admission to predict AKI occurring within 5 days after ICU admission. The area under curve (AUC) and the 95% bootstrap confidence interval were calculated and performed. We determined independent predictors of mortality/non-renal recovery using univariable and multivariable logistic regression with the same variables. The initial multivariable model was started as previously described with the same parameters. PC and sTM were forced in the model if their interaction term was in the model. Appropriate transformation was applied when needed and all statistical tests were two-sided and p<0.05 was considered significant. Statistical analyses were performed with IBM SPSS Statistics 19.0 (IBM Corporation, Armonk, NY) and R Core Team (2013). R: A language and environment for statistical computing. R Foundation for Statistical Computing, Vienna, Austria.

## Results

### Enrollment

**Fig 1:**
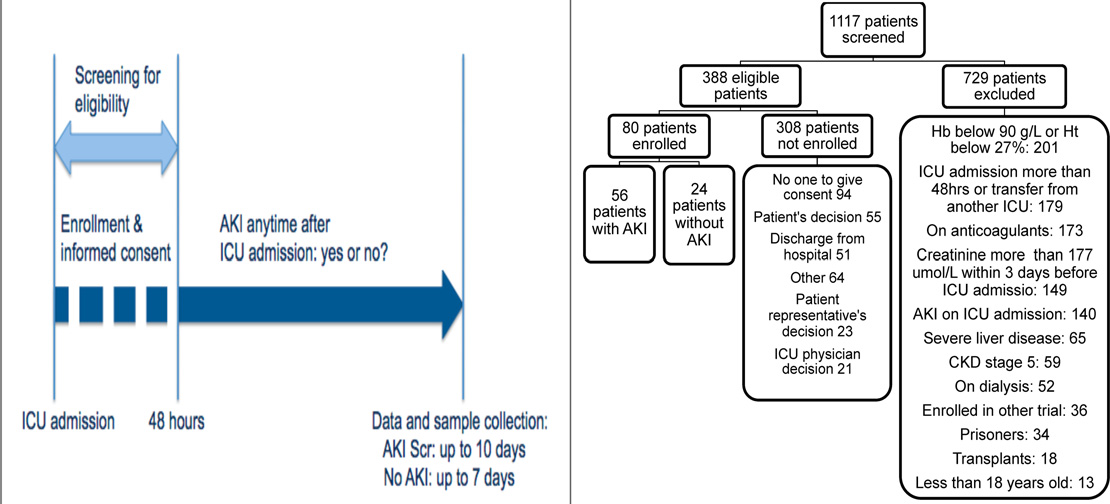
a and b. Screening, enrollment procedures and follow-up of study patients. AKI: acute kidney injury, AKI Scr: acute kidney injury based on serum creatinine criterion, CKD: chronic kidney disease, Hb: hemoglobin, Ht: hematocrit, ICU: intensive care unit. Reasons for exclusion can include more than 1 criterion.

Between July 2006 and December 2008, 1117 patients admitted to the ICU were screened for eligibility, and 388 patients were eligible. Among these patients, 80 were enrolled in the study (Fig. 1a). The reasons for non-enrollment are shown in Fig. 1b.

### Baseline characteristics

Baseline characteristics are shown in Table 1. The mean age was 56.9±15.7 years; 53.7% were male; 63.8% were white, 17.5% were black, and 12.5% were hispanic. At ICU admission, Scr and blood urea nitrogen (BUN) levels were 84 (IQR 71–106) μmol/l and 5.0 (IQR 3.0–8.0) mmol/l, respectively and 2.5% had severe oliguria, defined as UO <400 ml/24 hours. At ICU admission, 35.0% of patients required mechanical ventilation, 10.0% were on vasopressors, 42.5% underwent surgery, 7.5% had sepsis and APACHE III and SOFA scores were 23.0 (IQR 13.5–43.5) and 3.5 (IQR 1.3–6.0), respectively.

**Table 1:** Characteristics of the study patients at intensive care unit admission.

Characteristic	AKI according to serum creatinine (n = 18)	AKI according to urine output alone (n = 38)	No AKI (n = 24)	P
Age (years)	63.8 ± 13.3	56.6 ± 17.1	52.2 ± 13.5	0.06
Sex (%)				0.35
Male	10/18 (55.6)	23/38 (60.5)	10/24 (41.7)	
Female	8/18 (44.4)	15/38 (39.5)	14/24 (58.3)	
Race (%)				0.51
White	10/18 (55.6)	26/38 (68.4)	15/24 (62.5)	
Black	4/18 (22.2)	4/38 (10.5)	6/24 (25.0)	
Hispanic	2/18 (11.1)	5/38 (13.2)	3/24 (12.5)	
Other/unknown	2/18 (11.1)	3/38 (7.9)	0	
Weight (kg)	75.1 (IQR 63.0–86.6)	77.0 (IQR 64.8–90.9)	67.9 (IQR 65.4–77.3)	0.46
CKD status	4/17 (23.5)	0/38 (0)	3/24 (12.5)	0.11
Baseline eGFR (ml/min/1.73 m^2^)	60.1 (IQR 45.4–82.1)	85.9 (IQR 59.1–105.2)	89.7 (IQR 72.1–104.0)	0.04
Hypertension	12/18 (66.7)	18/38 (47.4)	10/24 (41.7)	0.26
Diabetes	11/18 (61.1)	8/38 (21.1)	6/24 (25.0)	0.007
CHF	9/18 (50.0)	3/37 (8.1)	2/22 (9.1)	<0.001
Cirrhosis	2/18 (11.1)	2/38 (5.3)	1/23 (4.3)	0.43
Creatinine* (μmol/l)	110 (IQR 95–143)	79 (IQR 62–97)	75 (IQR 64–88)	<0.001
BUN (mmol/l)**	8.4 (IQR 5.4–12.3)	5.1 (IQR 3.0–7.6)	4.0 (IQR 2.9–5.1)	<0.001
Urine output (ml/24 hrs)	722 (IQR 393–1523)	758 (IQR 444–1151)	1440 (IQR 875–2282)	0.001
Sepsis	2/18 (11.1)	1/38 (2.6)	3/24 (12.5)	0.18
On vasopressors	3/18 (16.7)	4/38 (10.5)	1/24 (4.2)	0.42
On mechanical ventilation	5/18 (27.8)	14/38 (36.8)	9/23 (39.1)	0.77
SOFA score	3.5 (IQR 1.75–5.25)	3.5 (IQR 0–6)	6 (IQR 2-)	0.72
APACHE III score	38.0 (IQR 18.5–43.5)	23.0 (IQR 15.0–59.0)	13.0 (IQR 13-)	0.69
Site				0.02
UCSD	8/18 (44.4)	26/38 (68.4)	15/24 (62.5)	
University of Alabama	10/18 (55.6)	7/38 (18.4)	9/24 (37.5)	
Université de Montréal	0/18 (0)	5/38 (13.2)	0/24 (0)	
Type of ICU				0.02
Medical	15/18 (83.3)	17/38 (44.7)	13/24 (54.2)	
Surgical	3/18 (16.7)	21/38 (55.3)	11/24 (45.8)	

AKI: acute kidney injury; ICU: intensive care unit; CKD: chronic kidney disease; eGFR: estimated glomerular filtration rate; CHF: cardiac heart failure; BUN: blood urea nitrogen; SOFA: Sequential Organ Failure Assessment; APACHE: Acute Physiology and Chronic Health Evaluation

AKI according to serum creatinine is defined as an increase in serum creatinine level of more than 27 μmol/l or more than 50% from a reference creatinine within 48 hours with or without urine output less than 0.5 ml/kg/hour for at least 6 hours (AKIN criteria)

AKI according to urine output alone was defined as urine output less than 0.5 ml/kg/hour for at least 6 hours without significant changes in serum creatinine

No AKI was defined as no significant changes in serum creatinine or urine output

*to convert from μmol/l to mg/dl, divide by 88.4

**to convert from mmol/l to mg/dl, multiply by 2.81

Among the 80 patients, a total of 56 patients (70%) developed AKI; 18 patients (22.5%) according to Scr criterion (AKI Scr) and 38 (47.5%) according to UO criterion alone (AKI UO). AKI occurred after a median of 1.0 (IQR 0–2.3) days following ICU admission for AKI Scr and 0.9 (IQR 0–2.5) days for AKI UO criterion. AKI based on AKI Scr was attributed to acute tubular necrosis in 28% of patients, associated cardiovascular events in 17%, unknown/undetermined factors in 38% and 17% of patients were considered to have multiple contributing factors. Patients who developed AKI Scr had higher BUN and creatinine at ICU admission and were suffering from more comorbidities than non-AKI patients (Table 1). They also had greater in-hospital mortality (AKI Scr 27.8% vs. 0% for AKI UO alone vs. 1.6% for no AKI, p<0.01; AKI Scr and AKI UO alone p<0.001 and AKI Scr and no AKI p = 0.008) (Table 2). However, the hospital length of stay was similar between the groups (AKI Scr 5.5 (IQR 4.0–14.8) vs. AKI UO alone 6.0 (IQR 4.0–13.0) vs. no AKI 5.0 (IQR 3.0–12.0) days; p = 0.24). There were no differences in terms of dialysis-dependence or renal recovery among groups.

**Table 2:** Outcomes of the study patients.

Outcomes	AKI according to serum creatinine (n = 18)	AKI according to urine output alone (n = 38)	No AKI (n = 24)	P value
**AKIN stage 1 (%)**	17/18 (94.4)	8/38 (21.1)	0/24 (0)	<0.001
**AKIN stage 2 (%)**	1/18 (5.6)	21/38 (55.3)	0/24 (0)	
**AKIN stage 3 (%)**	0/18 (0)	9/38 (23.4)	0/24 (0)	
**Mortality at hospital discharge (%)**	5/18 (27.8)	0/38 (0)	1/24 (4.2)	0.001
**Dialysis-dependence in survivors at hospital discharge (%)**	0/13 (0)	0/38 (0)	0/23 (0)	NS
Absence of renal recovery at hospital discharge* (%)	1/12 (8.3)	1/38 (2.6)	1/23 (4.3)	0.69
**Hospital stay (days)**	5.5 (IQR 4.0–14.75)	6.0 (IQR 4.0–13.0)	5.0 (IQR 3.0–12.0)	0.39

*absence of renal recovery defined as a difference between creatinine at hospital discharge and reference creatinine >27 μmol/l in survivors

### Patterns of sTM and PC levels in relation to AKI status

**Fig 2:**
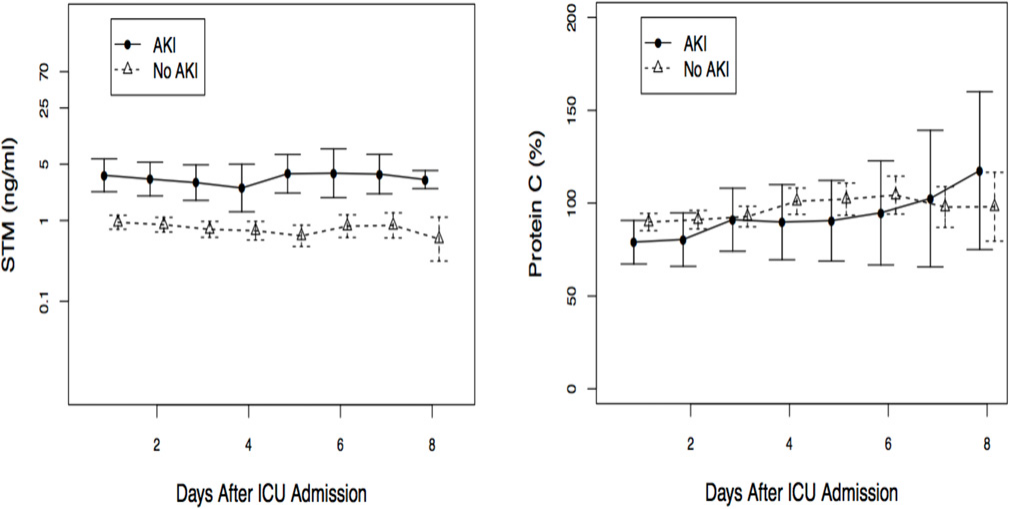
a and b. Mean daily soluble thrombomodulin and Protein C levels stratified by acute kidney injury status following ICU admission. Acute kidney injury status is based on serum creatinine criterion. These are means with 95% confidence interval.

Over the entire study period, sTM levels were higher in patients who developed AKI Scr (n = 18 patients) compared to patients without AKI Scr (n = 62 patients) (Fig. 2a). However, PC levels were similar between the AKI Scr and non-AKI groups (Fig. 2b).

**Fig 3:**
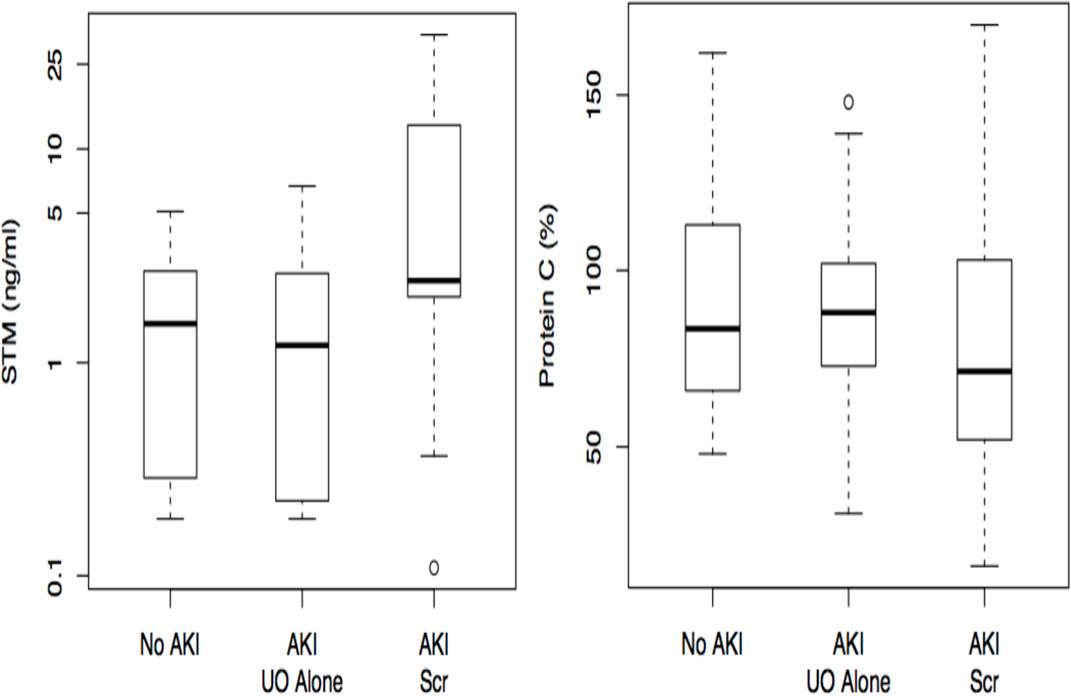
a and b. Median soluble thrombomodulin and Protein C values over the first three days according to AKIN diagnosis criteria. Soluble thrombomodulin: AKI Scr: 3.06 ng/ml (IQR 1.69–10.2 ng/ml), AKI based on urine output alone (UO): 1.35 ng/ml (IQR 0.36–2.47 ng/ml), No AKI: 0.42 ng/ml (IQR 0.22–2.39 ng/ml), Significant differences between categories: p<0.001, No acute kidney injury (AKI) (n = 24) vs AKI UO alone (n = 38) p = 0.84, No AKI (n = 24) vs AKI serum creatinine (SCr) (n = 18) p<0.0001, AKI UO alone (n = 38) vs AKI SCr (n = 18) p<0.0001. Protein C (p = 0.15): AKI Scr: 83.0% (IQR 50.0–118.0%), AKI UO alone: 87.0% (IQR 72.3–103.0%), No AKI: 92.5% (IQR 72.3–117.8%).

As AKI is an early event, we then assessed the differences in marker levels over the first three days after ICU admission for AKI Scr, AKI UO and no AKI. STM were significantly higher for AKI Scr compared to AKI UO and no AKI (Fig. 3a) whereas there were no differences between PC levels between AKI Scr, AKI UO and no AKI (Fig. 3b).

For other commonly used biomarkers, such as serum and urine NGAL and cystatin C, as well as urine KIM-1 and L-FABP, the difference in markers levels were not significant between AKI Scr, AKI UO and no AKI over the first 3 days. Since biomarker levels were similar between AKI UO and no AKI, in subsequent analyses we have looked at the differences between patients with AKI Scr (n = 18) and the remaining patients without AKI (n = 62).

### STM and PC levels in relation to the timing of AKI diagnosis

**Fig 4:**
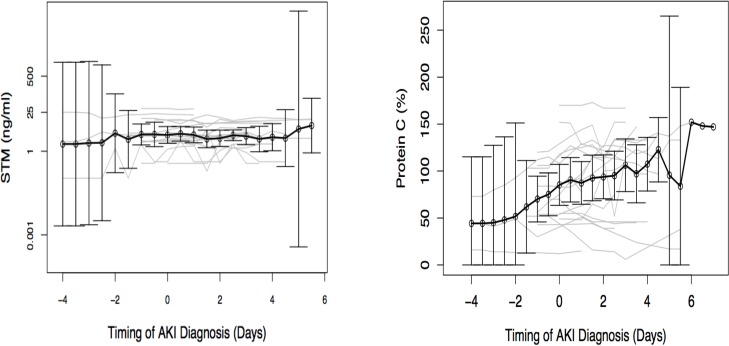
a and b. Mean soluble thrombomodulin and Protein C levels in relation to the timing of acute kidney injury diagnosis. Acute kidney injury diagnosis is based on serum creatinine criterion and occurred on day 0. These are means with 95% confidence interval.

We assessed the relationship of changes in marker levels to the timing of AKI Scr (Fig. 4a and 4b). STM levels were higher two days before AKI Scr and slightly declined thereafter. STM levels were significantly higher 24 hours before AKI Scr in the AKI group compared to the first 24 hours after ICU admission in patients without AKI (3.53 ng/ml IQR (2.16–9.81 ng/ml) vs. 1.25 ng/ml (IQR 0.22–2.61 ng/ml); p = 0.002). Similarly, PC levels were lower 24 hours before AKI Scr diagnosis in the AKI group compared to PC levels over the first 24 hours after ICU admission in patients without AKI, although this result was not statistically significant (56.0% (IQR 47.0–104.0%) vs. 86.0% (IQR 70.0–102.0%); p = 0.11). PC levels were below normal range (<70%) until 24 hours before AKI and levels increased progressively after AKI diagnosis (Fig. 4b).

To assess the value of sTM as a potential biomarker for AKI Scr, we computed the area under receiver operating characteristic curve (AUC) using ICU admission biomarker value to predict AKI within 5 days after ICU admission. The AUC was 0.77 (95% CI, 0.62–0.89). We also computed the AUC for PC to predict the *absence of* AKI within 5 days after ICU admission (as higher values of PC would theoretically “protect” from AKI and lower values would theoretically be “predictive” of AKI). The AUC was 0.63 (95% CI, 0.44–0.78).

We also computed the AUCs of other biomarkers to predict AKI, namely Scr, serum and urine NGAL and cystatin C, as well as urine KIM-1 and L-FABP (Table 3). STM levels predicted AKI Scr better than serum and urine NGAL and cystatin C, urine KIM-1 and L-FABP.

**Table 3:** Area under the curves of kidney biomarkers to predict acute kidney injury.

	AKI Scr (n = 18) vs. all no AKI Scr (n = 62)
Creatinine	0.90 (95% CI, 0.74–1.00)
Protein C	0.63 (95% CI, 0.44–0.78)
Log10 sTM	0.77 (95% CI, 0.62–0.89)
Log10 serum NGAL	0.59 (95% CI 0.37–0.80)
Log10 urine NGAL	0.67 (95% CI 0.51–0.85)
Log10 serum cystatin C	0.71 (95% CI 0.31–0.87)
Log10 urine cystatin C	0.72 (95% CI 0.53–0.87)
Log10 urine KIM-1	0.67 (95% CI 0.51–0.82)
urine L-FABP	0.61 (95% CI 0.46–0.77)
Log10 APACHE III	0.67 (95%CI 0.54–0.79)
Sepsis	0.56 (95%CI 0.46–0.67)

AKI Scr is defined as the AKIN serum creatinine criterion

### Factors predicting AKI

We then performed a time-dependent Cox regression proportional hazard analysis including all biomarker values as well as APACHE III score and sepsis status. Serum NGAL was not included in the initial multivariable model due to its high correlation with urine NGAL. In the final multivariable model, only PC and urine NGAL were independent predictors of AKI (Hazard ratio (HR) 0.98 95%CI 0.96–1.00, p = 0.04 and HR 2.36 95%CI 1.12–4.95, p = 0.02, respectively).

### PC and sTM levels in relation to mortality and/or non-renal recovery

**Fig 5:**
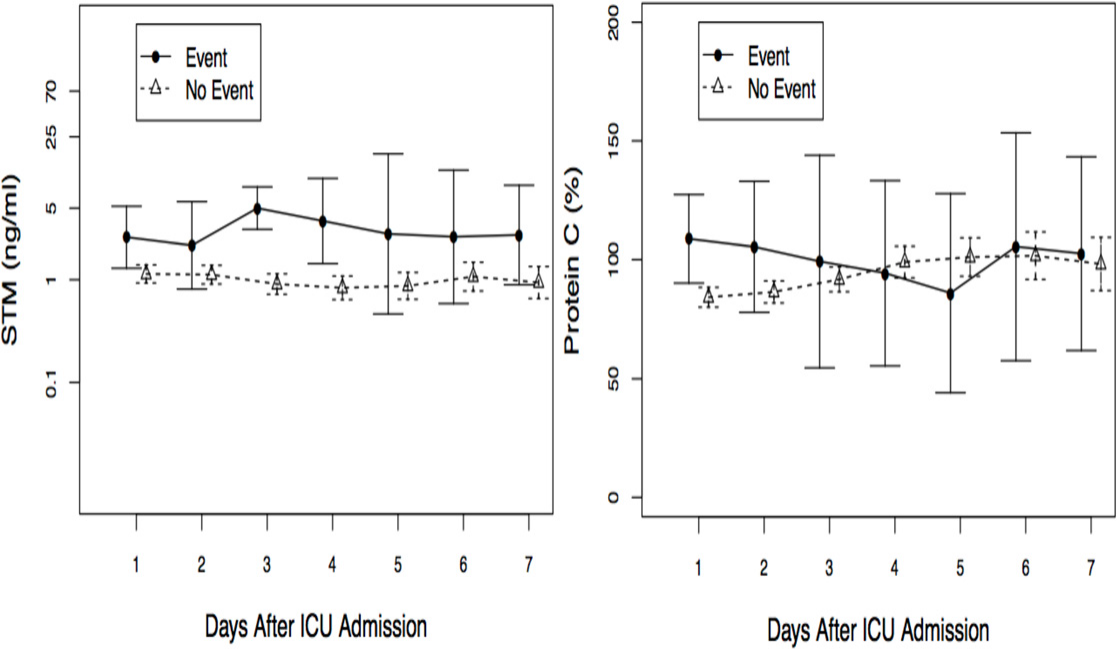
a and b. Mean soluble thrombomodulin and Protein C values stratified by all cause hospital mortality or non-renal recovery. Event means all cause hospital mortality or non-renal recovery. These are means with 95% confidence interval.

Soluble thrombomodulin and Protein C values stratified by all cause hospital mortality or non-renal recovery are shown in Fig. 5a and 5b. We performed a univariable logistic regression with the same variables to predict a combined outcome of mortality or non-renal recovery. In this model, all variables except serum NGAL were included in the initial multivariable model due to its correlation with urine NGAL. In the multivariable model, only PC levels were independently associated with mortality and/or non-renal recovery (OR 0.97 95%CI 0.94–1.00, p = 0.04).

## Discussion

The role of endothelial dysfunction in critically ill patients is currently not well characterized and there are limited data on sTM and PC levels in human AKI. In our study, we found that sTM levels were significantly higher before AKI and declined thereafter to levels comparable to those without AKI. This finding persisted throughout the study period. In a heterogeneous critically ill population, sTM levels at ICU admission predicted AKI better than more commonly used biomarkers such as NGAL or cystatin C, KIM-1, or L-FABP [20]. These results support previous clinical studies showing that sTM levels are predictive of AKI in trauma patients.

In our study, PC levels were independent predictors of AKI and mortality/non-renal recovery, even after adjustment for sepsis status and severity of illness. In addition, we have shown a temporal relationship between the nadir of PC levels and AKI diagnosis. PC levels remained below normal range until 24 hours before AKI and increased substantially after AKI diagnosis. Over the last decades, PC levels have mostly been assessed in ICU patients with sepsis and septic shock, where they were significantly below normal range [21–24]. In comparison to our study, the PROWESS study included patients with severe sepsis and organ failure for at least 24 hours, accounting for the lower PC levels observed at baseline [24]. In a subset of patients who developed AKI, PC levels at baseline were not different compared to patients without AKI [12]. This lack of difference could be explained by the higher severity of illness scores, as recent observational studies have shown that organ failure might be a better determinant of PC levels than sepsis status [9,25]. In addition, the PROWESS study did not include sequential data on biomarker levels as our study. One clinical study, involving surgical critically ill patients, showed that low PC correlated with organ dysfunction and that PC levels decreased as the maximal renal SOFA score increased [9]. Another single-center study found that low PC and high sTM levels at hospital admission were associated with increased AKI in trauma patients [10].

PC and sTM levels may help to determine the pathways involved in AKI, compared to KIM-1, NGAL and L-FABP, which are more site-specific damage markers. In contrast, Scr and cystatin C are representative of alterations in glomerular filtration rate. In our cohort, there were no significant changes in PC and sTM levels in AKI patients with AKI UO alone. These results suggest that oliguria may represent various phases of AKI reflecting the underlying pathophysiology. For instance, auto-regulatory changes can result in a reduced urine flow, which are potentially reversible, whereas oliguria may also occur due to underlying kidney damage. In these situations, combining measurements of PC and sTM levels and other damage biomarkers with changes in Scr and cystatin C could facilitate the differential diagnosis of AKI. For instance, if PC and sTM are not changed, oliguria may represent a functional (pre-renal) state and suggest that endothelial dysfunction has not occurred. More studies are needed to better define the relationship between markers of endothelial dysfunction and AKI.

This study has several strengths. We included patients from three different centers in North America with different demographics and clinical conditions, to increase the reproducibility of our results in patients from other centers. We enrolled all patients before AKI diagnosis to optimize the capacity of the biomarkers to predict AKI and included patients with and without sepsis to increase the generalizability of our results. All patients had detailed data on AKI status according to Scr and UO criteria, sepsis status as well as severity of illness scores. Of note, very few studies have included AKI UO criteria [26]. We excluded patients treated with activated PC or warfarin, or who had severe liver disease to avoid interference with PC levels. There are also limitations. We screened a large number of patients to enroll 80 patients. The major limitations to enrollment in eligible patients were the absence of a proxy for consent and patients’ and physicians’ decisions not to participate. These results can be useful for planning future studies on biomarkers in critically ill patients. Second, we excluded patients without significant CKD, in whom biomarker levels may differ [27]. In addition, we did not assess two biomarkers recently approved by the Food and Drug Administration for AKI diagnosis, namely insulin-like growth factor-binding protein 7 and tissue inhibitor of metalloproteinases-2 [28–30].

Although our study has limitations, it is important to highlight the relationship between sTM, PC levels and AKI. We showed that sTM levels at ICU admission predict AKI in a heterogenous critically ill population and that they tend to peak 48 hours before AKI diagnosis based on Scr. PC levels were independent predictors of AKI and mortality/non-renal recovery, even after adjustment for sepsis status and severity of illness. Both markers levels were not significantly modified with oliguria alone, suggesting that endothelial dysfunction is not as involved in this setting. The timely use of these markers of endothelial dysfunction might be useful to predict AKI and warrants further studies to assess their potential contributing role in clinical AKI. Further studies in large cohorts are required to further confirm these findings.
